# The role of social innovation in tackling global poverty and vulnerability

**DOI:** 10.3389/fsoc.2023.966918

**Published:** 2023-03-21

**Authors:** Jeremy Millard, Vincenzo Fucci

**Affiliations:** ^1^Third Millennium Governance, Ry, Denmark; ^2^International Center, Danish Technological Institute, Taastrup, Denmark; ^3^Turin School of Development, International Training Centre of the ILO (ITCILO), Turin, Italy; ^4^International Institute of Social Studies (ISS), Erasmus University, Rotterdam, The Hague, Netherlands

**Keywords:** SDG1, COVID-19, social innovation, poverty, vulnerability, sustainable development, resilience, agency and structure

## Abstract

Tackling the rapid rise in global poverty is one of the most pressing challenges the world faces today, especially in this new age of turbulence. On top of the ongoing environmental crisis, the last fifteen years has been rocked by the financial crisis of 2007–8, compounded by the 2020 Covid-pandemic and then by the 2022 war in Ukraine, each of which has negatively impacted all aspects of sustainable development. Although in practice many development organizations have been using the methods and processes of social innovation to tackle poverty and vulnerability for many years, it is only recently that they have specifically begun to analyse and codify its contribution to these and other SDGs. Social innovation provides beneficial social outcomes for citizens and other actors, often at local level with the strong bottom-up involvement of civil society and through its cross-actor, cross-sector, cross-disciplinary and cross-cutting strengths. Importantly, it aims to empower those with a social need, particularly when they have little to begin with. It focuses on increasing the beneficiaries' own agency and capability rather than passively only relying on others to act on their behalf. This is done by transforming social relationships and developing new collaborative processes. Amongst a wide range of recent and contemporary sources, this paper analyses a large scale quantitative and qualitative global survey of social innovations that tackle poverty and vulnerability in different global regions. It examines various definitions of poverty, including extreme, absolute and relative measures as well as arguably more useful approaches like the Multidimensional Poverty Index. It proposes how social innovation should be recalibrated to meet the increasing threats of the new age of turbulence, including by deploying the sociological lens of the agency-structure dichotomy to show why the public sector needs to become involved more proactively in social innovation. It also looks at certain myths around poverty and vulnerability, examines why we need to revise our understanding of sustainable development and resilience, and why a new nexus approach is needed that combines SDG1 with other strongly related SDGs.

## 1. Introduction and literature review

This section undertakes a literature review of social innovation in the context of its relationship with sustainable development, poverty reduction and recent global changes. Additional relevant literature is examined at the end of this paper when discussing its results.

### 1.1. Social innovation and sustainable development

In the period since the 2008 global financial crisis and the UN's launch in 2015 of the 2030 Sustainable Development Goals (SDGs) for 2030 (United Nations, 2015), there have been increasing concerns about global development crises. Poverty, inequality, political instability, deteriorating security and mushrooming environmental threats have all risen to the top of the global agenda. Many of these societal challenges arise from so-called “wicked” problems, i.e., very complex and intertwined challenges which require the combination of highly differentiated types of knowledge and expertise, collaboration between multiple actors and an openness to new ideas and approaches. It is in such a space that social innovation has in recent years been recognized and able to thrive.

Social innovation is concept that has gained traction over recent years both in public debate and academia, especially in the social sciences. It is characterized by a mix of methodological meanings and many adaptations to different fields and disciplines and is considered a cross-cutting term around which it is difficult to put a hard boundary. As a result, there is a rather florid literature on the definitions, processes and main actors of social innovation. It has been described as “*...innovative activities and services that are motivated by the goal of meeting a social need and that are predominantly developed and diffused via organizations whose primary purposes are social*” (Mulgan et al., 2007). It is a process aimed at achieving “…*the satisfaction of alienated human needs through the transformation of social relations: transformations which “improve” the governance systems that guide and regulate the allocation of goods and services meant to satisfy those needs, and which establish new governance structures and organizations…*” (MacCallum et al., 2009). Such forms of innovation are “…*new ideas (products, services and models) that simultaneously meet social needs (more effectively than alternatives) and create new social relationships or collaborations. In other words, they are innovations that are not only good for society but also enhance society's capacity to act*.” (BEPA Bureau of European Policy Advisers., 2010).

For the purposes of this paper, the definition of social innovation is derived from a theoretical frame developed by the SI-DRIVE project partners (including the first named author of this article) funded by the European Union (EU), 2014–18 (https://www.si-drive.eu). “*Social innovation focuses on new social practices defined as a new combination or new configuration of social practices in certain areas of action or social contexts, prompted by certain actors or constellations of actors in an intentional targeted manner with the goal of better satisfying or answering needs and problems than is possible on the basis of established practices; at the end socially accepted and diffused (partly or widely) throughout society or in certain societal sub-areas, and finally established and institutionalized as social practices*” (Howaldt et al., 2014).

In this context, social practices are defined as everyday practices, such as going to work, purchasing and consumption habits, lifestyles, pastimes, personal and community relationships, etc., that are meaningful to people as part of their everyday life activities. If these become widespread in a given locality, group or population, they can be said to become typically and habitually performed in (much of) society, and thus become institutionalized, at least in the informal sense. During the preparation and early rollout of the SDGs, policy makers began to understand that historically all human development has relied on changing social practices and cultures. At this time the UN first explicitly recognized that social innovation, as a body of knowledge, theories and practices, is a mainstream tool for delivering sustainable development (UNDP United Nations Development Programme, 2014). Previously, development agencies rarely, if ever, used the term “social innovation” to describe many of the actions and processes of development. Today, however, the role of, especially, bottom-up social innovation in designing and delivering public services to income-poor and marginalized people in a gender sensitive manner, especially when based on local acceptance and advocacy campaigns, is seen as an important means of achieving the SDGs by 2030 (For example, United Nations, 2020).

There is an increasing convergence between the means and the ends of sustainable development and social innovation. Both are predicated on changing social practices in response to societal needs and the extent to which they ultimately become institutionalized and made routine. Such change can be encouraged through top-down processes or developed perhaps more slowly, and initially more informally, from the bottom through ordinary people's everyday ways of living and working adapting to their changing needs and environments. Social innovation is increasingly recognized as an important component of the new innovation framework necessary for sustainable development. For example, Millard (2021) shows that “*the concepts, methods and tools of social innovation are almost uniquely well suited to successfully support all the SDGs including in the post-COVID landscape*”. Fucci (2022) core argument is that there is a “*nurturing relationship between social innovation and sustainable development*”, and that all the “*requirements which are fundamental to the achievement of sustainable development are already embedded in social innovation*.”

Two main conclusions can be drawn from the current situation. First, it is clear that social innovation has made, and continues to make, an essential contribution to all aspects of sustainable development, not least due to the wider recognition of the need for much greater focus on inter- and transdisciplinary studies given that real people in the real world lead interdisciplinary lives. Indeed, social innovation tends to be much better than many other types of innovation, such as technology and business innovation, at cutting across and linking between the various dimensions of sustainable development (Millard, 2017a). Social innovation's focus on people's actual problems and opportunities on the ground, as well as directly involving and empowering all actors including the beneficiaries, obliges it to take a multi-dimensional view.

Second, the world has changed quite dramatically over the last two years. The COVID-19 pandemic has resulted in the worst global economic recession since the 1930s, dramatically shifting the perceptions of politicians, researchers, businesses and citizens alike, and brought the visibility of sustainable development center-stage. This has since been turbo-charged by the Ukraine war that is leading to even greater numbers of poor and vulnerable people and dramatically ramping up the prices of basic goods like food and energy. Societies around the world appear to be entering an age of more or less constant uncertainty and disruptive change, so need to become much more resilient in the face of existing and likely future shocks and emergencies. These will probably include further pandemics, the climate crisis, biodiversity loss, geo-political, trade and governance challenges, as well as critical socio-economic conditions like uneven growth, poverty, inequality, exclusion and the disruptive role of technology. Given that all such shocks are interrelated, social innovation's cross dimensional and inclusive nature is ideally suited as a mainstream resilience tool.

### 1.2. Social innovation and ending poverty in all its forms everywhere

In this context, this paper addresses SDG1 aiming to end poverty in all its forms everywhere. Its objectives include ensuring that the entire population and especially the poorest and most vulnerable have equal rights to economic resources, access to basic services, property and land control, natural resources and new technologies (United Nations, 2015). In this sense, SDG1 can be seen as the key baseline SDG acting as a necessary, though typically not a sufficient, condition for all other SDGs. Thus, SDG1 underlies many other SDGs in terms not only of financial poverty but also vulnerability, marginalization from mainstream society, deprivation, exclusion and diminished opportunities, for example as experienced by women, minority and other groups.

According to United Nations (2021), priority actions on poverty eradication include:

- Improving access to sustainable livelihoods, entrepreneurial opportunities and productive resources
- Providing universal access to basic social services
- Progressively developing social protection systems to support those who cannot support themselves
- Empowering people living in poverty and their organizations
- Addressing the disproportionate impact of poverty on women
- Working with interested donors and recipients to allocate increased shares of ODA (Official Development Assistance) to poverty eradication
- Intensifying international cooperation for poverty eradication.

Of particular relevance when contrasting different parts of the world, as in this paper, is the different formal definitions of poverty, normally as either absolute/extreme or relative. Absolute poverty measures the amount of money necessary to meet basic needs such as food, clothing, and shelter. Both the United Nations and the World Bank use the international absolute standard of extreme poverty set at the threshold of $2.15 per day in 2017 prices. The concept of absolute poverty is not concerned with broader quality of life issues or with the overall level of inequality in society. The concept therefore fails to recognize that individuals have important social and cultural needs. This, and similar criticisms, led to the development of the concept of relative poverty which defines poverty in relation to the economic status of other members of the society. In the EU, this is defined as people falling below 60% of median income in a given country who are formally classified as being at-risk-of poverty. Thus, people are poor if they fall below prevailing standards of living in a given society which means they are unable to participate in the mainstream activities of their community. Both absolute and relative poverty also reflect failures in society for redistributing resources and opportunities in a fair and equitable manner. This leads to deep-seated inequalities and thus to the contrast of excessive wealth concentrated in the hands of a few while others are forced to live restricted and marginalized lives, even though they are living in a rich economic area.

However, an important criticism of both types of poverty definition is that they are purely concerned with income and consumption (Sachs, 2005; Ravallion et al., 2009). A related critique is provided by Sen (1985) who understands economic growth and individual income “*as means to expanding freedoms*” of members of society and defines development as a “*process of expanding real freedoms*” of individuals as an end in itself. According to Sen, freedom includes “*capabilities*” such as “*avoiding starvation, premature mortality and freedoms associated with being literate, being able to participate in political and social life*.” In this view, the assessment of development should consider both the development of individual capabilities and the expansion of freedoms. Sun thus perceives poverty as the “*deprivation of basic capabilities rather than merely lowness of incomes*”, an approach which might be seen as a precursor to social innovation's focus on directly involving and empowering all actors including the beneficiaries themselves.

More recent critiques argue for bringing concepts of class back into poverty discussions by getting away from rather arbitrary absolute or relative poverty lines that distinguish between the “poor” and the “non-poor” as this can mask poverty's root causes by decontextualizing poverty from its political and economic context. This approach contends that the focus on poverty measurement based on changes in PPP income alone “*obscures historical capitalist accumulation processes (such as dispossession, proletarianization and depeasantization)*” suggesting a need “*to recenter the analysis on the material causes of poverty, which are rooted in the functioning of the capitalist system, its antagonistic character, and the class-based contradictions of production itself* ” (Özgün and Dolcerocca, 2023).

### 1.3. Global poverty before and since 2020

**Figure 1:**
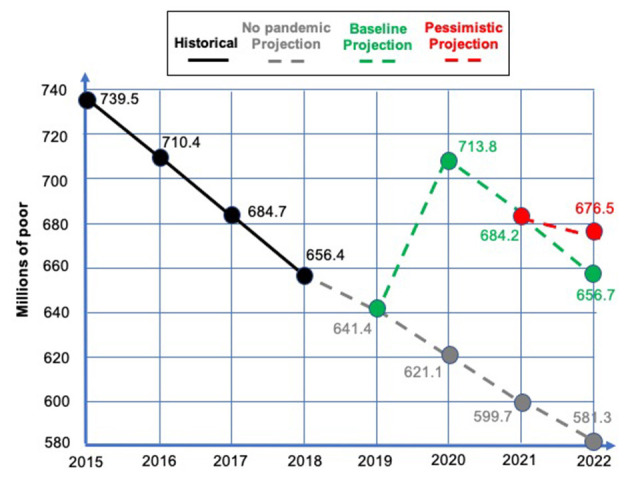
Extreme poverty, 2015–2022 (adapted from World Bank., 2022).

Tackling global poverty is one of the most pressing challenges the world faces today. Despite the significant global falls in extreme poverty during the fifteen years preceding the start of the COVID-19 pandemic, baseline projections for 2020, 2021, and 2022 again show dramatic increases, as illustrated in Figure 1. According to the United Nations (2022), COVID-19 has led to the first rise in absolute poverty in a generation. An additional 119–124 million people were pushed back into extreme poverty in 2020, and the global poverty rate is projected to be 7% in 2030, thereby missing the target of eradicating poverty.

Despite the success prior to COVID-19, relative poverty in Europe has also since risen threefold, so that in 2020, 96.5 million people in the EU27 were at risk of poverty or social exclusion, equivalent to 21.9 % of the population. Even more significantly, in almost all European countries, income as well as social and other forms of inequality rose significantly (Eurostat, 2021). This explains the sharp rise in relative poverty as the top 5% of the population pull increasingly away from the other 95%, both in terms of their financial assets and in the quality and security of the lives they lead. In Oxfam. (2020) claimed that “the world's richest 1% have more than twice as much wealth as 6.9 billion people”.

Other indicators that look at poverty from different perspectives also show a deteriorating situation. The global Multidimensional Poverty Index (MPI) released by the UNDP United Nations Development Programme (2021) and the Oxford Poverty and Human Development Initiative offers a clear picture especially in the most vulnerable countries. It measures poverty by taking into account and assessing distinct forms of deprivation suffered by people in their daily lives, including poor health, inadequate education and low standards of living. Across the 109 countries studied, the 2021 report shows that 1.3 billion of almost 6 billion people covered are affected by multidimensional poverty, with nearly 85% living in sub-Saharan Africa or South Asia and half under age 18. Among the main deprivations are out-of-school childhood, lack of basic assets, lack of drinking water within a 30-min roundtrip walk, shortage of electricity, undernourishment, exposure to solid cooking fuels, inadequate sanitary facilities and sub-standard housing. Key findings of the disruptive impact of the pandemic show an increasing level of inequality among countries in dealing with COVID-19. Indeed, in high-MPI countries, emergency social protection has been very limited, the share of employed non-wage workers has dramatically grown and many children have had to stop attending formal education (UNDP United Nations Development Programme, 2021).

### 1.4. Aim and structure of the paper

The main aim of this paper on the role of social innovation in tackling global poverty and vulnerability is to simultaneously better understand how the poor, vulnerable and marginalized can be better empowered to participate in meeting their own social and other needs, whilst at the same time addressing the structural and contextual barriers preventing them from doing so. After this introduction, the materials and methods deployed in helping to address this aim are outlined, followed by the presentation of some results from a global survey of social innovations that tackle poverty and vulnerability in different global regions. The final section discusses and analyses the main results, relates this to other evidence, examines the challenges social innovation faces in addressing poverty and vulnerability in the new age of turbulence and proposes ways in which social innovation needs to recalibrate to meet these challenges.

## 2. Materials and methods

This paper draws on a large scale quantitative and qualitative survey of social innovation around the world undertaken by the European SI-DRIVE research project, 2014–18 (https://www.si-drive.eu), supplemented and updated by studies and insights over the last 3–4 years during times of dramatic and often turbulent change.

### 2.1. Conceptual framework

SI-DRIVE developed a conceptual framework around five key dimensions that were operationalized as a basis for collecting data and analyzing social innovations around the world (SI-DRIVE., 2018):

1. Concepts and understandings: including social practices, social demands, societal levels and innovation dynamics: covered in the social practices and societal level and innovation dynamics sections.
2. Governance in terms of actors, sectors and actor roles: covered in the actors section.
3. Resources, including people and finance: covered in the resources section.
4. Drivers and barriers: covered in the drivers and barriers section.
5. Process dynamics phases, including the scaling and transfer and development paths: covered in the scaling and transfer and modeling development paths sections.

### 2.2. Sample and data collection

**Figure 2:**
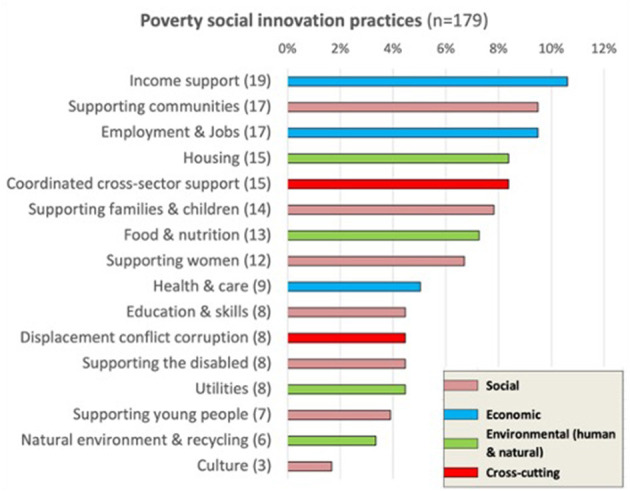
Social innovation practices aimed at reducing poverty and vulnerability.

SI-DRIVE's approach was based on a comprehensive review of literature and liaison with other social innovation projects preceding two rounds of global mapping. First, to collect 1,005 detailed case studies using both quantitative and qualitative methods, and second to carry out in-depth mainly qualitative investigations of 82 of these cases based on numerous interviews, background research, as well as data from the round 1 mapping. Poverty reduction and sustainable development was one of seven policy fields investigated (the others being: education; employment; environment; energy supply; transport and mobility; and health and social care), contributing 179 cases in round 1 and thirteen cases in round 2. Based on data from the 179 cases, cluster techniques were used to identify sixteen different clusters of social practice in the poverty policy field each with its own specific objectives to meet a specific social need (see Figure 2). The thirteen round 2 cases for in-depth analysis were selected to obtain wide geographic coverage and a balance in other case characteristics like size, scope and actors involved. The main conceptual frame for analyzing the cases were the five key dimensions mentioned above.

In practice it is impossible to undertake a fully statistically significant representative and comprehensive sample of social innovation cases given its definition is not generally fixed amongst practitioners and across countries and that many take place under the radar and without those involved necessarily using this terminology. Thus, the approach adopted was to establish a partner network of 16 EU and 9 non-EU researchers and practitioners around the world, each with intimate knowledge of social innovation, both in their own as well as neighboring countries and regions which didn't have their own partner (See list of partners in the acknowledgments below). For the purpose of this survey, all partners agreed to use the working definition of social innovation as given in the social innovation and sustainable development section, as well as the five dimensions as given above for structuring the questionnaires, thereby enabling the analysis to undertake relevant comparisons. Based on this and their specific country or regional knowledge, each partner identified a number of different types and sizes of initiatives that represented the status of social innovation in their country/region that were:

- Live (or recently completed) with at least one year of live operation
- Relatively successful, or likely to be successful, in terms of moving toward achieving some or all of their objectives, making it possible to analyse good practices with a focus on what works
- Prepared to complete a detailed quali-quantitative questionnaire and to be interviewed.

In this way, it was possible to compile a large dataset of 1,005 social innovation initiatives from around the world that have, or were likely to have, some success and which were able to report a large amount of both quantitative and qualitative globally comparative information suitable for scientific analysis.

### 2.3. Data analysis

Based both on the 179 questionnaire responses for the poverty and vulnerability reduction cases as well as 826 non-poverty cases, comparative analysis was undertaken using IBM SPSS and MS Excel software, mainly using Chi-Square analysis, Student's t tests and One-Way ANOVA. In the results section of this paper, data is only presented in the figures when it is statistically significance at the *p* < 0. 05 level. For the 13 qualitative in-depth interviews, a number of comparative workshops to consolidate findings were held with the partners responsible, who were also able to contact relevant cases again with follow-up questions or to clarify issues. In addition, NVivo word clustering software was used to undertake textual analysis in order to supplement and validate appropriate inferences derived from the quantitative data and to identify common themes and evidence-based insights.

## 3. Results

### 3.1. Social practices and societal level

As illustrated in Figure 2, analyzing and clustering the 179 poverty cases shows that income support (e.g., micro-financing and financial safety nets), supporting communities and employment and jobs are the three most common social practices in the poverty policy field. Also important are housing, coordinated cross-sector support (including between diverse actors and institutions), supporting families and children, combatting inadequate nutrition and hunger, and supporting women. Further analysis enables these practices to be grouped to reflect the three dimensions of the UN's framework for sustainable development: economic, social and environmental. This was done by cross-comparison with the texts of the UN's Agenda for Sustainable Development (United Nations, 2015) and specifically in relation to these three dimensions examined in this and related UN documents. A fourth cross-cutting dimension is also added because many social innovations aimed at people in the poverty policy field focus on more than one of the three dimensions at the same time. Defining and identifying both the most common social practices and then allocating them into these four groups was also validated by discussion with all partners and advisors (see the acknowledgment section below for a list of these). The social needs addressed by the poverty cases illustrate their huge range and diversity, given that they cut across all the other six more sectorally and specifically focused policy fields defined by SI-DRIVE, mentioned above and collectively defined here as non-poverty cases. This demonstrates that efforts to tackle poverty and vulnerability affect all aspects of life and society so that breaking down sector and actor silos is necessary to achieve success.

**Figure 3:**
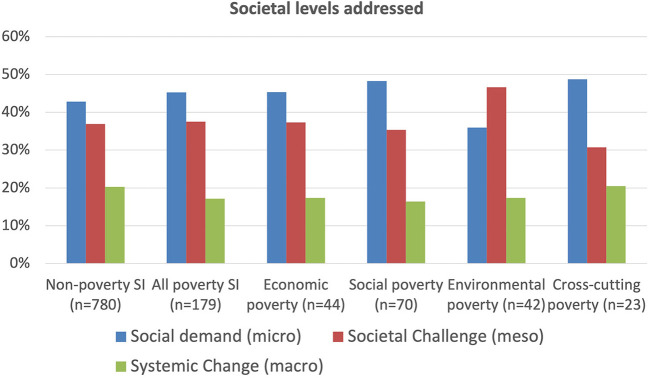
Societal levels addressed.

Social innovation for poverty is also distinctive from the non-poverty policy fields in terms of the societal levels addressed, as illustrated in Figure 3. Three societal levels are identified: (1) social demand (micro) tackling the immediate challenges of individuals and communities normally at a small scale; (2) societal challenges (meso) tackling challenges across society as a whole; (3) systemic change (macro) tackling challenges by changing the fundamentals of society (BEPA Bureau of European Policy Advisers., 2010; Hochgerner, 2013). There is a tendency for poverty social innovations to focus more strongly on the short term, micro and local social demands that poor and marginalized people face, compared with non-poverty social innovations which, as mentioned above, are highly focused on a particular sector like education, health, transport, the environment, etc. This is probably because their needs are typically more immediate and serious than those of the rest of the population. Poverty social innovations have a similar focus on the meso level societal challenges as for non-poverty initiatives, but its short-term focus is at the expense of the longer term more macro need for systemic change.

Also shown in Figure 3 is the breakdown of the poverty social innovations into four groups reflecting the three dimensions of the UN's sustainable development framework (economic, social, environmental) with the additional fourth cross-cutting dimension in which a case focuses strongly on at least two of the other dimensions simultaneously. Each group includes a number of specific social practices, as shown in Figure 2, where it is clear that the micro level is more important for social and cross-cutting practices. However, of interest, is that the cross-cutting group is, at the same time, the one most concerned with systemic macro change given that it needs to address, for example, silo-thinking in public governance and establish links between different sectors in attempts to maximize longer-term resilience.

### 3.2. Innovation dynamics

**Figure 4:**
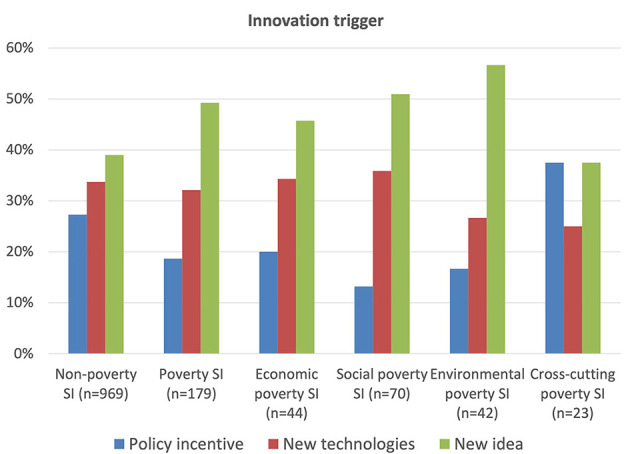
Innovation trigger.

Data on the triggers of both non-poverty and poverty social innovations in Figure 4 clearly show that the most important triggering mechanisms are new ideas, but much more so for poverty cases, probably due to their more recent appearance and thus the relative dearth of existing good practices to learn from and/or adopt. New technologies are the next important trigger, but more so for non-poverty cases where especially ICT and social media are more widely used. Significantly, policy innovations are generally less prominent in poverty cases, perhaps again reflecting their more recent appearance and the challenges in tackling poverty issues. This is especially the case given they are more likely to take place very locally driven by civil organizations, as shown in the next sub-section, reflecting that poverty social innovations are more likely to be on a small scale and often under the radar not influenced by top-down policies. As regards the four poverty groups, policy incentives are much more important in the cross-cutting group, perhaps because these do, in fact, need to take account of the more complex and comprehensive nature of such cases that typically need to tackle policy barriers and involve policy actors. These cases tend to require good cooperation from the public sector through cooperation across government entities and policies which enable more personalized services and treatments. The environmental group is the most likely to be triggered by a new idea than any other type of case as these, even amongst poverty cases generally, are normally very recent and thus have much less precedent to work with in relation to issues like climate change, pollution, food quality and similar.

**Figure 5:**
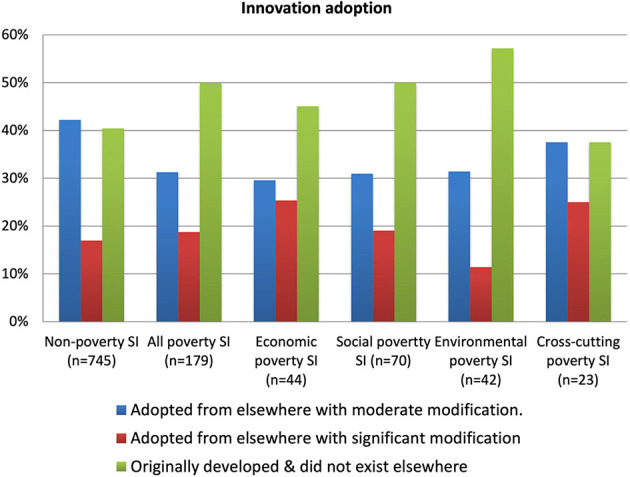
Innovation adoption.

Some similar conclusions are seen in Figure 5 regarding how the innovation was originally developed. While most non-poverty social innovations have been adopted from elsewhere, poverty cases are much more likely to be highly original social innovations which did not previously exist. This perhaps again reflects the fact that they tend to be more recent than non-poverty cases, to be under the radar and thus less influenced by external factors and, as shown by other data, more likely to be in developing and emerging economy countries where fewer good practices exist and the mechanisms for disseminating them are weaker.

### 3.3. Actors

**Figure 6:**
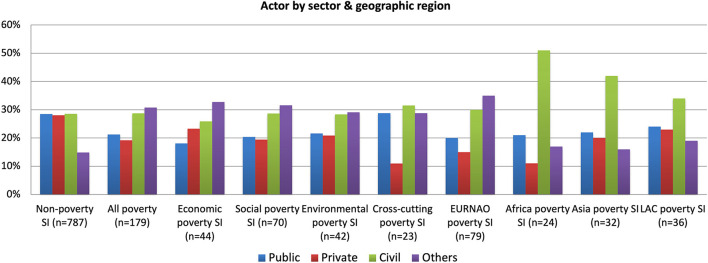
Actor by sector and geographic region.

As shown in Figure 6, all social innovations reveal strong collaboration between the public, private and civil sectors. However, poverty cases show that civil society organizations together with “other” actors, such as foundations, informal groups, schools, charities, religious groups and cooperatives, are relatively more important than public and private actors. Poverty cases collaborate much more with these “other” actors perhaps due to their near and local focus and more intense working with the beneficiaries themselves, which many of these “other” actors directly represent. These patterns are reflected in the four poverty groups, but where again cross-cutting cases stand out as involving relatively more public actors, given the need to address governance issues, and relatively less the private sector, whilst the reverse is the case with the economic cases.

There are also important differences between different geographic regions in Figure 6, so that the EURNAO region (Europe, North America and Oceania, i.e., generally the more developed countries) are much more likely to involve “others” in the actor mix. This is perhaps related to the relatively more strongly developed ecosystem of such actors than elsewhere. In contrast, in poverty cases in Africa, Asia and LAC (Latin America and the Caribbean) countries, it is civil society organizations that lead poverty social innovations but supported by a correspondingly less developed ecosystem of “other” actors.

**Figure 7:**
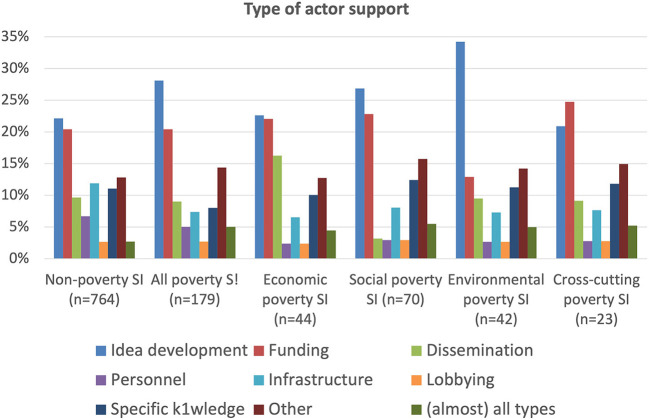
Type of actor support.

Figure 7 displays the various ways in which actors provide support to the different types of case, with idea development and funding roles as the most important for both non-poverty and poverty cases. There are also distinct differences, so that idea development is even more important in poverty cases, which also rely more on both “other” and “(almost) all types” of roles, so are more wide ranging. This is perhaps because poverty social innovations tend to be relatively more recent than other types and are more likely to need a richer and diverse ecosystem of actors and inputs to succeed. Indeed, the data overall show that poverty cases have a more multi-varied character than non-poverty cases, and that their ecosystems tend to be more diverse, rich and broad. Within the poverty cases, the environmental group has the most prominent role for idea development, maybe reflecting the increasing urgency being placed on good ideas to counter climate change. This role is also relatively more prominent for the social group, perhaps because of perceptions that concrete impacts are more difficult to achieve and take longer than in other groups, so that the onus on innovative ideas is greater. Funding tends to be the biggest type of support in cross-cutting cases, probably because, to be successful, they need strong commitment from funders due to the need to marry both the very local needs of beneficiaries with the more societal level governance and structural challenges.

**Figure 8:**
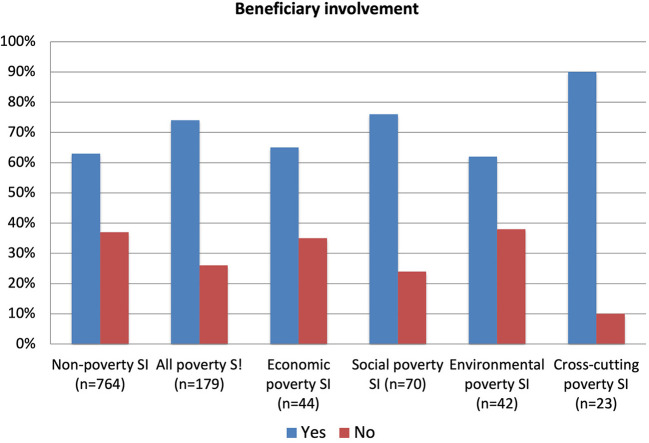
Beneficiary involvement.

An important characteristic of social innovations generally, in some contrast to more technology and business focused innovations, is their very strong emphasis on involving the beneficiary as much as possible in both designing and implementing the innovations that will directly affect their lives. Figure 8 shows that 63% of non-poverty social innovations have direct beneficiary involvement, but that this proportion increases to 74% with poverty cases. The clear conclusion is that these social innovations targeting the poor, marginalized and vulnerable, focus even more than other social innovations on the direct involvement of beneficiaries. The data also shows that, within all poverty cases, the cross-cutting sub-group is even more focused on involving the beneficiary with 90% of such cases doing so. This probably refers to in-kind direct support from the beneficiaries themselves, who tend to get more directly involved in co-creating and running such initiatives, given the need for these to be highly personalized around their unique integrated needs as individuals or groups. A strong characteristic of poverty, as compared to non-poverty, social innovations is that they have multiple affects across most or all aspects of the individual's life. This means they need to take these all-round needs into account if they are to be successful, a situation seen most strongly in the cross-cutting cases.

### 3.4. Resources

**Figure 9:**
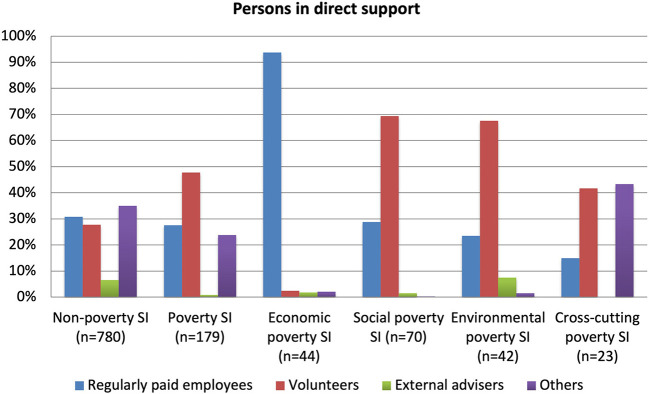
Persons in direct support.

The most important resources for any type of social innovation tends to be people and finance, but there are sharp contrasts between the different types. In terms of people, Figure 9 shows that non-poverty compared to poverty cases have a more overall balanced array of personnel between regularly paid employees, volunteers, external advisers and others. The relative importance of external advisers is very low in both types but virtually absent in the poverty cases which rely much more on volunteers. This can likely be explained by these social innovations generally being less well established and professional and, as shown by other data, being more common in developing and emerging economies than non-poverty initiatives. This is not the case, however, for the economic poverty group which uses very few volunteers but has huge reliance on regularly paid employees, which is very likely because of the need for more personnel from the economics profession and from the private sector.

**Figure 10:**
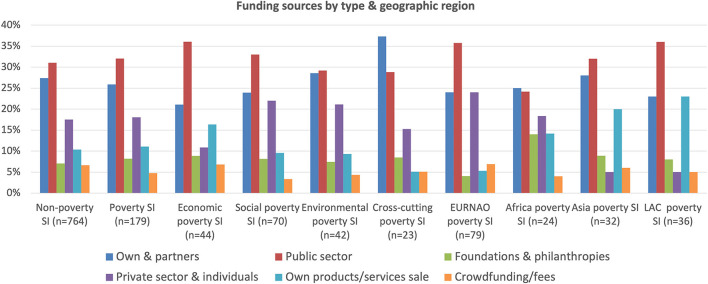
Funding sources by type and geographic region.

The relative importance of different funding sources, shown in Figure 10, reveals much similarity between both non-poverty and poverty social innovations, as well as across all types of poverty cases. All draw upon on a wide range of types, with the most important typically being public sector, own and partner contributions. In addition, private company and private individual funding are important, as is the sale of own products and services. Other data show that non-poverty cases are more likely to draw on public EU funding where they are able to do so, whilst in contrast foundations and philanthropies provide relatively more funding in African, Asian and LAC countries, where these organizations have even greater focus on poverty and vulnerability than elsewhere.

Both the environmental and cross-cutting poverty groups rely significantly more on their own and partner contributions, and correspondingly less on public funding, perhaps explained by the former's smaller scale compared with other poverty cases and the latter's more personalized and integrative nature. Poverty cases also use crowdfunding less than non-poverty cases, again given that there are fewer of these in the more developed economies where such finance tends to be more accessible. This is also seen in the geographic data for poverty cases where those in the EURNAO region rely more on crowdfunding than in the other regions. In contrast, obtaining funding from foundations and philanthropies and charging users for products and services is seen much more in African, Asian and LAC countries given the greater challenges they have in obtaining funding from the public and private sectors.

### 3.5. Drivers and barriers

**Figure 11:**
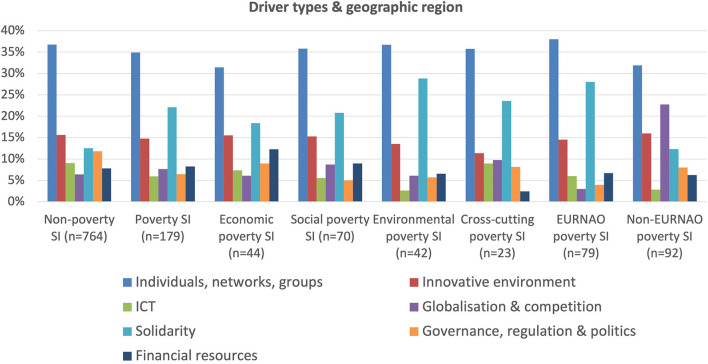
Driver types and geographic region.

As shown in Figure 11, by far the most important drivers of both non-poverty and poverty social innovations are relationships and interactions with individuals, networks and groups, plus, but to a lesser extent, an innovative environment and solidarity. However, ICT and social media are less important as a driver of poverty cases, whilst these have a much greater focus on solidarity, reflecting their attention on vulnerability and marginalization. The economic poverty group has somewhat less focus on solidarity and more on financial resources as would be expected, whilst the cross-cutting poverty group has the lowest focus on financial resources probably reflecting its need to focus more on governance, regulation and politics as it attempts to integrate different parts of the public sector to support beneficiaries' individual all-round needs.

Looking at differences between geographic regions for drivers and barriers, we have combined the African, Asian and LAC regions into a non-EURNAO region given the low amount of deviation between them. On this basis, the non-EURNAO regions are much less characterized by a vision of solidarity, perhaps because of the greater competition for resources and the difficulties in recognizing common needs. These regions are also markedly less likely to be driven by individuals, networks and groups or by ICT and social media, which probably reflects the large access, cost and skill differences between the two groups of countries, particularly when dealing with poor and marginalized people. The non-EURNAO region is also much more driven by globalization and competition given its greater exposure and sensitivity to such forces.

**Figure 12:**
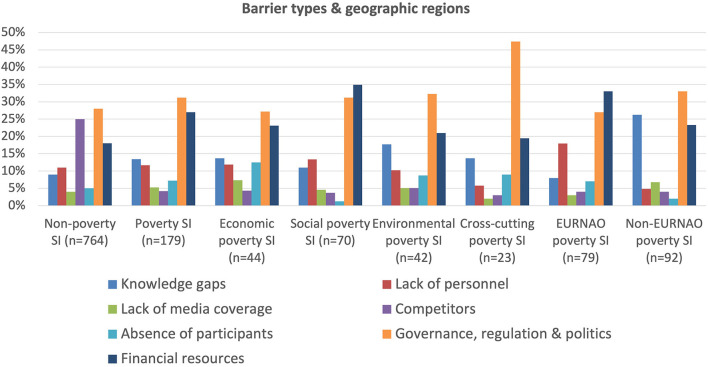
Barrier types and geographic region.

With regard to barriers to both poverty and non-poverty cases, Figure 12 shows that governance, regulation and politics is by far the most prominent given that these issues are traditionally not as conducive to social as they are to technology or business innovation, however here the similarities disappear. For non-poverty cases competition is the next barrier, followed by financial constraints. The presence of competitors is seen as a barrier in non-poverty cases as these cases are more sectorally focused, as for example in health, education, transport, etc., in which private sector businesses are much more likely to be competing against social innovations, most of which are not profit-seeking. In contrast poverty cases rarely meet such competition as businesses generally do not see these cases as opportunities to create profit. For poverty cases, the next most important barrier is financial constraints, probably both because these generally have greater challenges in attracting finance and that they are more common in the non-EURNAO countries. Knowledge gaps and lack of personnel are also important barriers to poverty social innovations.

Both the social and the cross-cutting poverty groups are more affected by governance and financial barriers than the economic and the environmental groups, both of which tend to be better able to attract business finance and government support. The cross-cutting poverty group is the most challenged by governance, regulation and political barriers, which may be due to the fact that it often requires a whole-of-government response which is often difficult given the siloed nature of many public sectors around the world.

In terms of differences between the EURNAO and non-EURNAO regions, it is clear that both lack of knowledge and governance barriers are much greater in the latter. In contrast, lack of finance is a bigger barrier in the EURNAO region perhaps because social innovations in these countries are traditionally more prone to use, and thus more sensitive to, financial inputs during the recent period of relatively austerity compared to before the 2007–8 financial crisis. Governance, regulation and political barriers are also more important in the non-EURNAO region, almost certainly due to greater scope here for conflicting interests around legality, legitimacy and power. This is, for example, illustrated by many cases in this region where social innovations supporting poor and vulnerable people often fill a gap where there are no appropriate public services. This can also mean that public authorities can become resentful by thereby being shown to not providing support in a context where, in principle, they have a duty to do so.

### 3.6. Scaling and transfer

**Figure 13:**
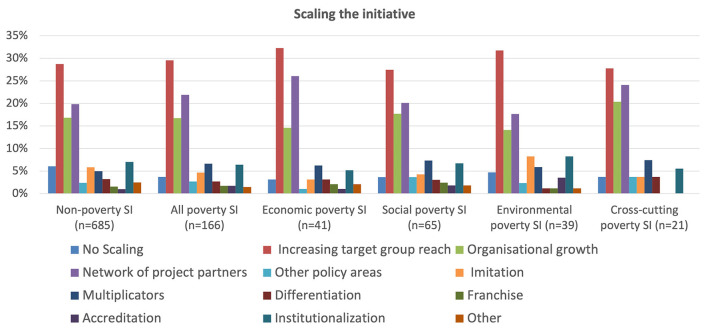
Scaling the initiative.

Scaling, as opposed to transfer, refers to a social innovation initiative growing *in situ*, e.g., when its own governance and organization grows organically and thereby itself serves an increasing number of users and beneficiaries. Figure 13 shows there are strong similarities between poverty and non-poverty cases, with the most important being increasing the target group reached, having a network of project partners and simple organizational growth. There are, however, some noteworthy distinctions amongst the poverty groups, such as the importance the economic group gives to its network of project partners for project scaling. This is probably because these cases are typically those with the longest pedigrees and that are most advanced and likely to be supported by the private sector. This also applies to some extent to the cross-cutting cases where the explanation is more likely to be these cases' greater diversity of partners, given their cross-cutting and cross-sectoral nature, so they potentially have more partner channels to work with.

**Figure 14:**
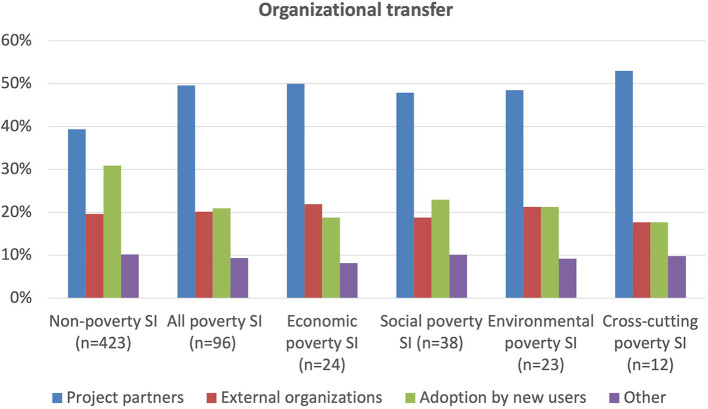
Organizational transfer.

Evidence for organizational transfer shows some clear distinctions between non-poverty and poverty social innovations, as shown in Figure 14. The latter are much more likely to be transferred by project partners themselves and less likely to be taken up by a new group of users. This almost certainly reflects the fact that poverty cases tend to be newer and less advanced and thus more likely to be known and appreciated only by a narrower group of actors, particularly of course the project partners themselves. There are also minor, but significant, differences between the poverty groups, so that economic cases more often transfer *via* external organizations that can be their social enterprise partners. Social cases, in contrast, are relatively more likely to attract new users perhaps because they involve a greater number of civil and “other” actors each of which is likely to bring many more users, as evidenced in Figure 6.

**Figure 15:**
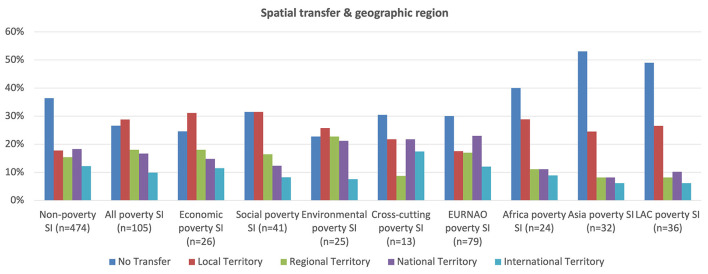
Spatial transfer and geographic region.

Figure 15 shows that the spatial transfer of social innovations provide some of the best direct evidence of successful case outcomes and impacts, given that, although cases can of course have great impact in their specific context even if not transferred, the fact of transference is a clear sign of success. Evidence of spatial transfer shows that although poverty cases are more likely to be transferred than non-poverty cases, they are much less likely to be transferred over a greater distance. This is probably because non-poverty cases tend, as noted above, to be more specialized and sectorally focused so are only transferred if highly similar needs and conditions arise elsewhere. However, if they do arise elsewhere with the necessary specialist, sectoral and governance conditions, transfer can take place relatively easily and over considerable distances. In contrast, poverty social innovations, being relatively more recent and less advanced than other social innovations, are nevertheless more likely to be transferred simply because there are fewer good practices to share so demand for them grows more rapidly from increasingly aware communities of policy-makers, funders and civil organizations.

The main differences amongst the poverty groups show that social cases are much more likely to be transferred over shorter distances with cross-cutting cases transferring over greater distances. This perhaps is because the former are more idiosyncratic and locally contextualized, whilst the latter's good practices, when successful, represent models of comprehensive and integrative innovation, bringing together diverse partners and interests the principles of which can be more readily transferred. Looking at differences between geographic regions, EURNAO poverty cases are much more likely to be transferred and spread over greater distances, which probably reflects their greater communication and networking resources and capacities.

### 3.7. Modeling development paths

**Figure 16:**
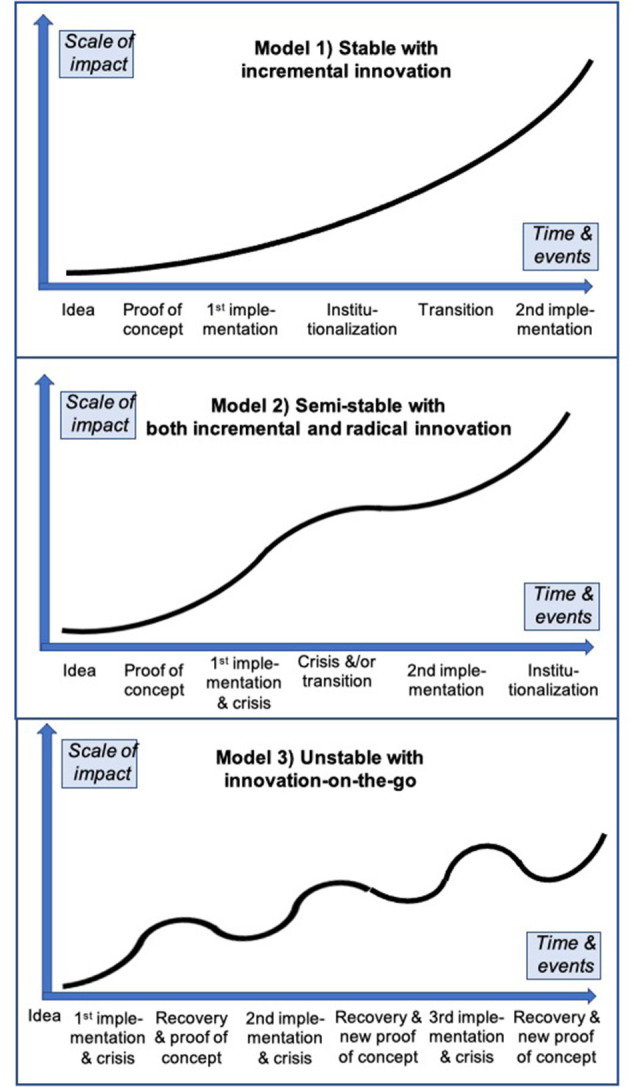
Three generalized development path models.

An examination of the large variety of development paths exhibited by social innovation initiatives for poverty and vulnerability reduction provides good evidence of how, and the extent to which, they interact with wider society. Drawing on this diversity, Figure 16 depicts three overarching generalized models that can be readily discerned in the 179 cases studied. This might also apply to many other types of social innovation, given that the poverty cases overlap significantly with the other SI-DRIVE policy fields (Millard, 2017b).

#### 3.7.1. Stable with incremental innovation

This type represents a quite stable, robust and a relatively closed top-down development path embedded in widely agreed policy and regulation, so has good efficiency and can be effective, and often characterized by incremental innovation given the general lack of serious challenges or crises. The typical development path of this type is for more or less steady and continuous growth, typically related to relatively large stable government and/or other funding and support within a conducive policy structure where the case objectives overall are meeting their intended outcomes.

This path is illustrated by the *Strengthening Popular Finances (Ecuador)* case as an example of income support practices. This initiative provides alternative financial services to a vulnerable rural population lacking access to commercial bank credit, in order to promote local development through the use of small remittances and savings. The Ecuadorian Populorum Progressio Fund (FEPP) identified the need for financial structures in which the community could place its savings and receive credits. The only credit channel previously available was the *agiotista* (loan sharks) who charge very high interest rates of up to 50% erecting a barrier to productive activities and the creation of new micro-enterprises. The main success factor is that the initiative is embedded in law and the constitution for receiving remittances directly from beneficiaries and customers, thereby eliminating intermediaries (including loan sharks and the banks). The case simultaneously also gives beneficiaries their own agency to act in future on behalf of themselves and others, especially through its many local networks linked together by a national umbrella network.

#### 3.7.2. Semi-stable with both incremental and radical innovation

This type successfully mixes both top-down and bottom-up methods and resource, typically quite stable at the macro level but less so at the micro level, both relatively open and closed, generally robust, relatively effective and can be efficient. It is often characterized by a mix of incremental and disruptive/radical innovations related to some, but relatively few, serious challenges or crises. The typical development path of this type is a step-by-step or stage model, characterized by two or three main stages separated by slower or no growth with many boosts punctuated by a major challenge or crisis which, if survived, becomes a turning point. This tends to be due to financial, political, personnel or other serious problems, albeit not existential, during which there is little or no direct support from policy structures or other supports, at least during the slow-down, but where the case objectives overall continue to meet their intended outcomes.

This path is illustrated by the *School for life (Ghana)* case as a Ghanaian NGO that developed a programme in rural northern Ghana to bring 'complementary basic education' to 8–14 year-olds from poor families who would otherwise not receive any schooling. Impressive results have been achieved since programme start in 1995, including over a half million girls and boys who are now literate. Over 5,000 “barefoot teachers” have also been trained, i.e., locally recruited people speaking the local language and with at least basic literacy and numeracy skills who receive a 6-week crash teaching course. This takes place in collaboration with a Danish NGO and the Ghanaian government funded by Danish and, later, other countries' aid money. Development has not been without several challenges, such as with the established teachers' union and, more seriously, with the Ghanaian government which feared the initiative would usurp their role of provider of basic education, thereby threatening the viability of the initiative on several occasions. However, once the government recognized the high value of the approach, it was adopted as official policy with the government taking some credit, leading to the innovation becoming increasingly institutionalized for hard-to-reach groups and locations.

#### 3.7.3. Unstable with innovation-on-the-go

This type tends to be loosely structured, relatively bottom-up and small scale, typically quite unstable due to fast changing conditions and more subject to tensions so needs to become shock resistant in order to achieve and succeed. These initiatives are often quite open, can be both relatively effective and efficient but also the reverse, often characterized by frequent disruptive (if not radical) innovation and “innovation-on-the-go” with both many boosts and crises. The typical development path is for up and down, wavelike, alternating success and failure, mainly due to very fast changing dynamic contexts directly affecting the social innovation but which the social innovation is attempting to address. In many such examples, the policy structures may be neutral but also sometimes negative although rarely consistently so at least over the longer term, and where the case objectives overall are meeting their intended outcomes.

This path is illustrated by the *Taste of home (Croatia) case that* draws on the specific cooking and gastronomic, as well as language, skills of refugees to create an environment for their economic emancipation as a part of their social inclusion and integration into the host society. Intercultural integration refers to the process of the social inclusion and economic emancipation of refugees and other persons with migrant backgrounds. It is an effort that seeks to provide a pathway both for the arriving and domestic population to interact in a positive shared atmosphere, as well as to enable the immigrants to develop marketable skills they can use to become full economic contributors and beneficiaries within Croatia. Given the variety of place-specific implementations in practice, and the fast-changing challenges, conflicts regularly arise for different reasons and with different outcomes, often due to tensions between displaced persons and the host societies. In this sense, conflict becomes a barrier to successful social innovations which need to respond in sensitive and constructive ways involving all actors but, if successfully negotiated, can become a strong mechanism of social change.

The second development model is the most common, but there are also many successful examples of the other two. It tends to be the most resilient as it is able to balance between the relative lack of innovative stimulus of the more top-down and stable regime, typical of the first path, on the one hand, against the relatively high instability and existential threats faced by the third path that can only be held at bay if the initiative can regularly find new ways to radically innovate, on the other. The success ratio of initiatives required to adopt the third model is less than the other two but this type is important especially in the new age of turbulence the world seems now to be entering. However, initiatives required to adopt the second model can have the greatest overall impact given there are more of them and that their relative stability itself increases impact whilst the fact that they still need to be nimble means they are unlikely to ossify or lose their innovative potential. Neither should the first path be dismissed given that, being closely aligned to prevailing structures and policies it can provide large scale impacts over short as well as long timescales, although the lack of challenges also means it is likely to lose its innovative edge. However, given that the long-term ambition of most social innovations is to become informally institutionalized as widespread social practices and even more formally institutionalized in existing structures, this model can also be a sign of great success. When this happens, new social innovations, and new types of social innovation, are likely to arise, perhaps adopting models 2 or 3, or devising completely new models.

## 4. Discussion

### 4.1. Highlight findings

The results above show that social innovations tackling poverty and vulnerability, much more than social innovations generally, are typically undertaken not only through important collaborations between the public and/or private sector, but also depend largely on the essential contribution of many non-mainstream actors. Specifically, these consist of civil society organizations and that miscellaneous macrocosm that is the third sector, including non-profit associations, community organizations, social enterprises, mutuals, charities and other similar socio-economic agents. They are bound together by a common vision of inclusion and solidarity, and this extends to the people actually experiencing poverty and vulnerability, so the incorporation of these beneficiaries into the process of social innovation is vital. This also helps to prioritize the coordination and integration of initiatives, given that vulnerable people typically experience multiple deprivation challenges that single-sector or single-actor interventions can often exacerbate rather than ameliorate due to siloed-thinking, siloed-practice and groupthink

Success is thus often dependent on cross-actor, cross-sector, cross-disciplinary and cross-cutting, bottom-up, small scale and highly local and contextualized initiatives. These typically work closely with the target beneficiaries to increase the latter's capacities, capabilities and knowledge about their own needs and how they can participate in achieving them. Advocating for the right to have their social needs met is often an important component, both vis à vis the government and other powerful institutions and organizations, but also within the community itself to raise their own awareness in order to take collective action. The results also show that, over the medium to longer-term, large successful social innovations often need to become widespread social practices which can be strongly boosted by “institutionalization”, i.e., accepted and adopted by these powerful institutions and organizations and perhaps even taken over, partially or fully, by them. The importance of this cannot be over-emphasized, as mentioned in the SI-DRIVE definition of social innovation given above (Howaldt et al., 2014). The question is, how can this be done?

In many poverty-focused social innovations, the key movers are civil organizations which are typically more trusted by the poor and vulnerable as they have greater local knowledge and are more nimble—they act, in effect, as “trusted third-parties”. This typically seems to work well given they are seen as not having their own commercial or political interests and are thus better able to be neutral mediators. For example, in the basic education, gender empowerment and employment for vulnerable communities case in northern rural Ghana, outlined above, a local NGO partially supported by Danish development funding, has successfully managed to mediate and coordinate appropriate solutions by combining the efforts and resources from a range of actors. These include both central and local governments, trades unions, local micro-enterprises, youth groups, radio and TV outlets, village chiefs and councils, with whole village communities often mobilized, as well as international donors and experts. A common feature of this and many similar initiatives is the adoption of all-round approaches addressing the whole human being with dignity and respect and within the context of transparent justice and the rule of law. According to Novogratz (2016) “*The opposite of poverty is not income, it is dignity*”, thereby echoing the critiques of mainstream poverty measurement approaches made, for example, by Sen (1985) and Özgün and Dolcerocca (2023) outlined in the social innovation and ending poverty in all its forms everywhere section.

This means that basic questions need to be asked about how social needs and issues are articulated. For example, on the one hand, the poor typically find themselves in a condition of overall relative powerlessness, whilst on the other hand the poor—and especially the communities in which they live—possess huge potential, resilience and latent ability to be a big part of their own solution. This will often mean there should be less focus just on “problem-solving” and much more on “opportunity seeking” by the poor in their specific context, so that open awareness raising, advocacy and mobilization of the poor and their communities, as much as possible through their own efforts, is critical. From the perspective of governments, funders and civil organizations, this implies that a coordinated cross-cutting approach is often needed which cuts across administrative silos and links together a range of complementary actors depending on the specific requirements of each initiative.

There are also important differences between social innovations tackling poverty in more developed countries and in developing countries. The former are more likely to involve a much wider range of actors drawn from strongly developed actor ecosystems, not just civil society organizations but also foundations, informal groups, schools, charities, religious groups and cooperatives. Initiatives in developing countries tend to be more recent and under the radar, where fewer good practices exist and mechanisms for disseminating them are weaker. They are also less characterized by a vision of solidarity and more subject to greater competition for resources, including financial and technological, reflecting their greater exposure to globalization forces. Governance, regulation and political barriers are also more important in developing countries, almost certainly due to the greater scope there is for conflicting interests around legality, legitimacy and power. There is greater need for social innovations targeting poverty in these countries to provide support and services to fill the gaps left wide open by both the public sector and the market when the latter do not provide such services. This can also lead to increased hostility from public authorities as any success a social innovation has in delivering what otherwise would be seen as basic public services, risks exposing these authorities' poor performance. This sometimes leads to the initiative experiencing increased challenges and crises, which can also mean they are less likely to be able scale or transfer over long distances.

Given that poverty both results from, as well as itself causes, multiple deprivation across a range of issues, this is a fundamental issue. Although social innovations targeting poverty and vulnerability focus strongly on the short-term more local, and often pressing, social needs of the poor and marginalized and that this is clearly important, it often does so at the expense of the longer term more systematic and structural changes needed in society which might alleviate these social needs in the first place. As indicated in Figure 3, most of the social innovation initiatives studied, especially those focused on poverty, are in essence concerned only to meet immediate social demands by supporting and increasing the agency, capabilities and empowerment of beneficiaries, without recognizing that typically these are merely the symptoms of more structural root causes, which are hardly considered let alone addressed. It is also clear that these social innovations need to be self-reflective regarding, for example, whose societal needs and challenges are being met by traditional practices and structures. Although this is absolutely essential, it means in turn that they are often very contextually-anchored and thus more difficult to transfer than many other social innovations, so either they do not transfer, or only do so over a short distance, as illustrated in Figure 15. Cultural, ethnic, and religious issues, as well as governance and institutional issues, can be decisive.

### 4.2. The challenges social innovation faces in addressing poverty and vulnerability in the new age of turbulence

As noted above and shown in Figure 1, poverty has increased dramatically since the start of COVID-19 after a period of decline. However, inequality and vulnerability have been rising in most countries since the 1980s, so that both the pandemic and the war in Ukraine can be interpreted as revealing and accelerating existing inequality trends. Especially since the 2008 financial crisis, taxation has become less progressive in many countries, whilst welfare states have become less generous and labor markets more volatile (Ortiz-Ospina and Roser, 2016). An increasing number of people work in informal, precarious or “gig” economy jobs, typically based on new digital technology, leading to increasing numbers of the “working poor”. Numerous developed economy governments have hailed their low unemployment rates as major achievements, but have sidestepped the issue of the poor remuneration, quality and security of many jobs. Many low paid and insecure workers experience poor, crowded and often unhealthy conditions prone to COVID-19 infection, but during the pandemic have been precisely those applauded by employers and governments as “essential workers” keeping the economy and society functioning. These include workers in the health and care sectors, transport and maintenance, cleaners and caterers, retail, food and agricultural workers, plus drivers and delivery workers, and many more (Fleming, 2020; Palomino et al., 2020).

These recent developments reveal another long-existing but largely ignored dilemma. The prime emphasis of poverty-focused, as well as other, social innovations is the attempt to improve the agency of vulnerable people so that they can increasingly address their own and similar social needs in future, whilst tending to ignore the wider societal structures which actually produce these social needs in the first place. Indeed, these structures are, by definition, beyond the control of the vulnerable who, in most cases, are not even aware of them. Social innovations, thus tend to focus on agency, empowerment, capability- and capacity-building, awareness-raising and advocacy, all of which are of utmost importance. They can be immensely successful and this clearly needs to be continued and strengthened. However, these qualities have limited degrees-of-freedom, and the pandemic, reinforced by the Ukrainian war, have revealed, not just a glass ceiling, but indeed many thick glass walls that severely constrain agency and significantly reduce its potential beneficial impacts. The barriers identified in Figure 12 are dominated by governance, regulation and politics, i.e., they are systemic and structural. This forces the typically bottom-up nature of most social innovations to focus mainly on what is possible under current conditions in the short-term, addressing the more local and often most visible social needs and/or the immediately realizable opportunities and low-hanging fruit. As shown in Figure 3, more than 80% of poverty-focused social innovations are aimed mainly at meeting micro, immediate or at best meso, medium-term social needs, with < 20% aiming to tackle more structural root causes at the systemic change level (see also Millard et al., 2018).

One of the most influential agency-structure studies recognizes that everyone has limited cognitive capacity and time, but the unique disadvantage of the poor and vulnerable is that they are typically pushed to and beyond these limits more than any other group. Mullainathan and Shafir (2013) show that the poor in any society have precarious structures within which to live and work. They literally expend all their effort simply surviving from day to day or week to week, and don't have sufficient time, energy or cognitive capacity to plan for and invest in their own, their family's or their community's, future. Being poor is literally a full-time and stressful occupation. This empirical research shows that when richer people are put in the same constrained conditions they react in the same way as the poor and often a lot worse. When any individual's cognitive capacity is strained in the way experienced by most poor people struggling to cope, it is equivalent to driving a car whilst drunk or reducing their IQ by 14%. Most poor people coping with these conditions are, in fact, performing extremely well just by surviving. They are far from being lazy, stupid or scroungers. This is not the traditional “poverty trap”, normally thought of as a self-reinforcing mechanism which sees the individual sink further into hopelessness through their own lack of effort to change their lives because of laziness or low intelligence. Instead, it recognizes that poor people, more than others in society, typically have to contend with a much more highly complex and unpredictable social and economic environment.

Mullainathan and Shafir (2013) undertook their research in the USA and India, respectively examining relative and absolute poverty but reaching similar conclusions in both regarding cognitive capacity and how individuals behave. McGarvey (2022) looked at relative poverty in the UK and recognized a “*proximity gap between the powerful and the powerless as the root cause of many of society's ills*.” In education, health, housing and the benefits system there is a fundamental and maintained distance between those who make policies and the people at the bottom of society who are on the receiving end of them. This is a gap in experience, understanding, culture and, above all, in empathy. McGarvey shows through his analysis of why and how a select group of policy-makers with very limited experience of social inequality has the power of discussing and debating it and making the rules and structures that surround the poor and vulnerable. The gap from those at the bottom of society, who live with the ramifications of these structures, policies and decisions, has been both revealed and turbo-charged during COVID-19. When social innovations focus all their energies only on the agency and capabilities of the powerless the results are likely to become largely meaningless akin to tackling only symptoms and not causes.

Another lesson of the turbulence arising from the pandemic and war in Ukraine is the need to re-focus the concept of societal sustainability toward a new approach to resilience. Among others, the WEF World Economic Forum (2020) suggests that, to better prepare for future shocks, especially including the climate crisis, short-term economic efficiency needs to be better balanced against longer-term resilience. This implies that the concepts of sustainable development require refocusing on this new balance in order, in WEF's words, to “build back better”. Economic efficiency has been the overriding mantra and has increasingly become purely a short-term goal, whilst economic and societal resilience requires a medium-to longer-term perspective which is also necessary in tackling poverty and vulnerability. To optimize sustainability, a trade-off is required between, first, efficiency which is necessary to ensure good use of limited resources but can lead to “brittleness” and low flexibility, and, second, resilience through diversity and interconnectivity which is adaptive to shock by providing some system slack (Millard, 2020). The “sweet spot” needs to be found between too much efficiency that risks large scale breakdown from time to time, and too much resilience which risks some stagnation. Most economies, whether developed, developing or emerging, have so far failed to achieve let alone recognize the need for such an “optimal balance” where both are in play. This is seen in the prevailing focus on short-term shareholder, as opposed to shared or stakeholder value (Porter and Kramer, 2011), “lean” companies and value chains and indeed “lean” governments (Millard, 2015). There has been an over-reliance on “just-in-time” governance and business models that allow little flexibility or ability to find alternative sources or processes in case of disruption, so that the need is now seen to be moving to “just-in-case” models and mindsets.

The economic mantra has been “squeezing assets” to near breaking-point, including human assets by pushing people into poverty and vulnerability, and tightly tuned value chains to maximize quarterly returns leaving no margin for flexibility or slack in case of shock. As a result of the economic and social disruptions caused by the pandemic and war, many international organizations and governments are starting to advocate building resilience much more strongly into economic systems at all levels. It is clear that resilience should be seen not only in narrow economic terms, but also as social and environmental resilience. This is the changing context of the new age of turbulence in which social innovation can recalibrate and thrive.

### 4.3. To tackle poverty and vulnerability in the new age of turbulence social innovation needs to recalibrate its goals and methods

Social innovation's important task in tackling immediate social problems and exploiting available low-hanging opportunities in order to curate the agency and capabilities of the poor and vulnerable is, as suggested above, only half the story. Especially as we enter the new age of turbulence, social innovation must also tackle the societal structures that determine and constrain this agency. There is a need to develop approaches, methods and structures to build between, and link, levels and actors, whilst focusing on real local issues. In an analysis of transformative social innovation for sustainable rural development, Castro-Arceab and Vanclay (2020) formulated the concept of “bottom-linked governance” as a multi-level middle ground linking bottom-up initiatives and top-down structures. This is where actors from various political levels, geographical scales and industry sectors come together to share decision-making. Social innovations tackling poverty and vulnerability have the potential to be transformative, but to do this, they need to scale-up from the local social demand level and provoke changes in the governance system at the systemic change level. Castro-Arceab and Vanclay (2020) identify bridging-role functions like network enabler, knowledge broker, resource broker, transparency and conflict resolution agent, and shared vision champion. A very similar approach is being piloted in the USA and UK under the label “community-wealth-building” that aims to link the different levels to support local development by attempting to retain as much as possible of the value generated locally within the locality. This is done, for example, by designating local anchor institutions (public, private and civil) that invest and procure locally where possible, and by increasing local, employee and citizen ownership and control (Democracy Collaborative, 2019).

Accordingly, structural readjustments in public governance, laws, regulations, cross-agency and non-government collaborations, institutional adaptation and innovation, etc., are needed. These should be designed to make the lives of the poor and vulnerable as easy and as simple as possible so that, in addition to direct support, they can focus on helping to solve their own actual problems of scarcity rather than grappling with a complex system that is often irrelevant to their particular context. Examples of such good top-down structural changes with clear success include the recent employment tribunal ruling in the UK that Uber can no longer treat its otherwise vulnerable drivers as self-employed, but instead as employees who have the right to receive the national living wage and holiday pay (Guardian Newspaper, 2021). This legal change considerably simplifies drivers' lives and provides them with more long-term security, with likely implications for the gig economy more broadly. An Indian example is the use of ICT to promote the financial inclusion of the poor by simplifying and linking up contextual structures and supports around them through the world's largest bio-metric ID system. This means that the pre-existing complex, siloed and separated system of subsidies and benefits for the poor are now provided through a one-stop shop with simple identification, both raising awareness of what the poor are entitled to and making it very easy to access their rightful benefits (Burt, 2019).

As these examples show, the government actor, and the public sector more generally, is often crucial in enabling social and other types of innovation to maximize their success through governance and structural, often, top-down changes. Especially governments need to provide or facilitate basic human services like education, health, social protection and utilities. Governments should promote human rights and be cast as “duty bearers” whilst the target group beneficiaries need to be seen as “rights-holders”. This is without denying that the latter group should also be subject to clear and specific obligations and duties, as indeed should all members of society. Indeed, this essential complementarity is emphasized by McGarvey (2022) when he calls for the “poor” to exercise the agency and capabilities they already have whilst simultaneously insisting that the state needs to make appropriate radical structural changes that can expand their degrees of freedom. Thus, although there is a strong need for government-led poverty reduction and welfare state policies, social innovators at all levels need to expand their activities. Both top-down structural changes and more bottom-up social innovation initiatives are necessary operating in combination.

Thus, beyond basic public services, governments are primarily responsible for good, open, transparent, inclusive, participative and ethical governance, including the legal and regulatory frameworks governing society. As noted by Mazzucato (2013), the “entrepreneurial state” needs to be pro-active and risk-taking, and not just correctors of market failure when this occurs. Governments are prime actors in tackling poverty and vulnerability, even when not being the most active or providing the most resources. They alone have the duty and the means to represent the interests of everyone in society and have the power and resources to determine the structures within which the society and economy functions. They need to work alongside and enable the social innovators, and often many others, through public, private, people partnerships (Millard, 2015).

Policy orientations for governments in this respect have been identified by the OECD (Bulakovskiy, 2021). Policy-makers should promote the creation of enabling environments and frameworks that foster the emergence and the development of more bottom-up social innovations through both demand and supply-side measures. On the demand side, the main goal would be creating a market for social innovation through a series of measures, including awareness campaigns and prizes, public procurement to prompt the integration of small social innovative enterprises, impact measurement tools to assess the relevance of such activities, and fiscal policies, namely tax incentives and subsidies. On the supply-side, these measures need to be intended to expand the number of actors and enhance the quality of their activities. These would include direct or indirect financing of social innovation initiatives, the provision of infrastructures allowing the environment to prosper, such as incubators, and the promotion of a process of skills development in accordance with the know-how required in the sector. Also needed is the provision of the suitable tools and means needed to foster effective cooperation with social innovators and practitioners.

In this context, the impact measurement issue for poverty-focused social innovation also needs overhauling. Whilst the more traditional, often quantitative, measures and logic models, should still be deployed where relevant, some of the techniques and approaches often used successfully by the international development community have an important role to play. These include the Multidimensional Poverty Index introduced in the global poverty before and since 2020 section, as well as: the “theory of change” which attempts to get away from path dependent thinking and traces the processes of how change actually happens in different contexts; “appreciative enquiry” which focuses not on solving a “problem” but on the capabilities and capacities already available or easily developed and how these can be used to identify opportunities for beneficial change; “outcome harvesting” that examines all actual outcomes, whether planned or unplanned, and then traces these back to see how they arose; and “key lines of enquiry” that focuses on monitoring key/desired issues like poverty reduction, gender, capacity building, etc.

Given social innovation's often unique capacity to break down silos and link across actors and sectors, it should be further enabled to develop new nexus combinations of SDGs to meet re-calibrated sustainability and resilience challenges, especially in the context of fighting poverty and vulnerability. Although the SDGs have already achieved a great deal, key aspects are failing (United Nations Human Rights Council, 2020), and there is widespread agreement of the need to recalibrate our response to COVID-19, the ensuing recession, and accelerating global warming. All SDGs are important, but tackling poverty and vulnerability underlies and can enhance the achievement of all others. Taking account of differences between developing and developed economies as outlined above, social innovation can assist SDG1 in developing nexus combinations in ways that are more relevant to real conditions on the ground than traditional approaches. Deploying a nexus approach is a recognition that any solution for a given problem must equally consider other issues in the nexus, for example in the water-food-energy nexus already formally established (United Nations Water, 2020). Nexus thinking has two key assumptions: a systems approach, where the interactions between different nexus components are taken fully into account, not just in the short-term but critically also over the longer-term; and a decision-making approach where policies, strategies and actions are based on these interactions within the nexus as a whole.

A new more formalized nexus combination, supported by social innovation, should be formed around poverty and hunger eradication and economic and gender equality. This could combine SDGs 1 (poverty), 2 (hunger), 3 (health and wellbeing), 4 (education), 5 (gender), 8 (inclusive and sustainable economic growth) and 10 (inequality) (Millard, 2021). These SDGs are typically interlinked on the ground reflecting multiple forms of deprivation and exclusion that include both extreme and relative poverty affecting peoples' lives even in developed countries. There is a need to focus on “all-round” approaches which treat people as whole individuals with their own dignities and identities. A poverty-vulnerability-exclusion nexus-thinking approach should be adopted to avoid focusing only on one part of the nexus without considering interconnections, risks and unintended consequences. Nexus thinking coupled with social innovation focuses on real life on-the-ground linkages, synergies and trade-offs to balance different interests and outcomes.

As argued above, harnessing social innovation to tackle poverty and vulnerability has traditionally turned a blind eye to prevailing political and socio-economic structures, largely accepting the latter as “given” and not directly relevant or useful to the ongoing practical on-the-ground work required to alleviate the problems being tackled, especially when these are urgent and sometimes immediately life-threatening. To some extent this is due to the relatively recent emergence of social innovation as a recognized and robust set of goals and methods for this purpose, as well as its locus largely in small-scale, often informal and under-the-radar, organizations and movements. Thus, even the rapid rise in poverty and inequality in the wake of the resurgence of neoliberalism in the 1980s through its “Washington consensus” focus on freeing-up markets from regulation and reducing the role of the state, as well as the more sudden crisis of the 2008 financial crash, tended to cement the largely bottom-up, here-and-now mindset of social innovation (Millard, 2014). The new set of global crises commencing in 2020, and seemingly ongoing, provides a new opportunity for social innovations tackling poverty and vulnerability to take a radically new, and arguably more mature and nuanced approach. This requires clear-sighted and painstaking work to combine social innovation's undoubted success in galvanizing the agency and capabilities of beneficiaries with the determination to achieve appropriate top-down structural changes and purposeful nexus partnerships to dramatically enlarge the degrees-of-freedom within which it operates. It is a case of “both, and” rather than of “either, or”.

## Data availability statement

The raw data supporting the conclusions of this article will be made available by the authors, without undue reservation.

## Ethics statement

Ethical review and approval was not required for the study involving human participants in accordance with the local legislation and institutional requirements. Written informed consent to participate in this study was not required from the participants in accordance with the national legislation and the institutional requirements.

## Author contributions

JM devised the overall approach and structure, analyzed and prepared the data and figures, and undertook most of the writing. VF provided sparring and a critical review, as well as contributed important texts. All authors contributed to the article and approved the submitted version.
